# The formation of volatiles in fruit wine process and its impact on wine quality

**DOI:** 10.1007/s00253-024-13084-8

**Published:** 2024-07-17

**Authors:** Jianxin Tan, Mingyue Ji, Jiangang Gong, Bimal Chitrakar

**Affiliations:** College of Food Science and Technology, Hebei Agricultural University, Baoding, 071001 People’s Republic of China

**Keywords:** Fruit wine, Fermentation, Volatile compound, Aroma profile, Quality

## Abstract

**Abstract:**

Fruit wine is one of the oldest fermented beverages made from non-grape fruits. Owing to the differences in fruit varieties, growing regions, climates, and harvesting seasons, the nutritional compositions of fruits (sugars, organic acids, etc.) are different. Therefore, the fermentation process and microorganisms involved are varied for a particular fruit selected for wine production, resulting in differences in volatile compound formation, which ultimately determine the quality of fruit wine. This article reviews the effects of various factors involved in fruit wine making, especially the particular modifications differing from the grape winemaking process and the selected strains suitable for the specific fruit wine fermentation, on the formation of volatile compounds, flavor and aroma profiles, and quality characteristics of the wine thus produced.

**Key points:**

• *The volatile profile and fruit wine quality are affected by enological parameters*.

• *The composition and content of nutrients in fruit must impact volatile profiles*.

• *Yeast and LAB are the key determining factors of the volatile profiles of fruit wines*.

**Supplementary Information:**

The online version contains supplementary material available at 10.1007/s00253-024-13084-8.

## Introduction

A fruit wine is an alcoholic beverage made by the fermentation of fruits, excluding grapes; the addition of flowers and herbs is sometimes involved. A fruit wine is usually named after the fruit from which it is produced; for example, blueberry wine is made from blueberries. These non-grape fruits from different geographical locations around the world used for producing fruit wines include apples, apricots, bananas, blackberries, blueberries, cherries, dates, hawthorns, kiwifruits, lemons, mulberries, and oranges (Swami et al. 2014; Jagtap and Bapat 2015; Saranraj et al. 2017; Velić et al. 2018a). Such winemaking process is mostly similar to that of grape wines, which mainly involves crushing to get fruit juice (also called a must); fermentation was conducted by yeasts along with or without other microbes, including lactic acid bacteria, maturation, clarification, aging, and bottling. During these processes, sugars and other nutrients in fruit musts are converted to alcohols, organic acids, esters, aldehydes, ketones, terpenes, phenolic compounds, etc. (Table S1), contributing to wine taste, flavor, and aroma (Carpena et al. 2020). Meanwhile, natural colorants, such as anthocyanins, chlorophylls, carotenoids, and bioactive compounds, are released in the wine to give it an attractive appearance and certain physiological activities (Kumar and Singh 2021; Nabi et al. 2023). All these metabolites generated from fermentation and maturation together with the nutrients originating from fruits determine the overall quality of fruit wines (Carpena et al. 2020). According to the recent year studies associated with the topic of fruit wine, this review will discuss the correlation among enological parameters, nutritional composition and content, and microorganisms involved in the formation of volatile compounds, flavor, and aroma profiles, ultimately affecting the quality characteristics of fruit wines. Given the diversity and characteristics of fruit wine raw materials, fruit wine differs from grape wine making processes in terms of particular modifications and selection of fermentation microbial strains, and their impact on the aroma profile of fruit wine is emphasized.

## Fruit wine making process and fermentation parameters

**Fig. 1:**
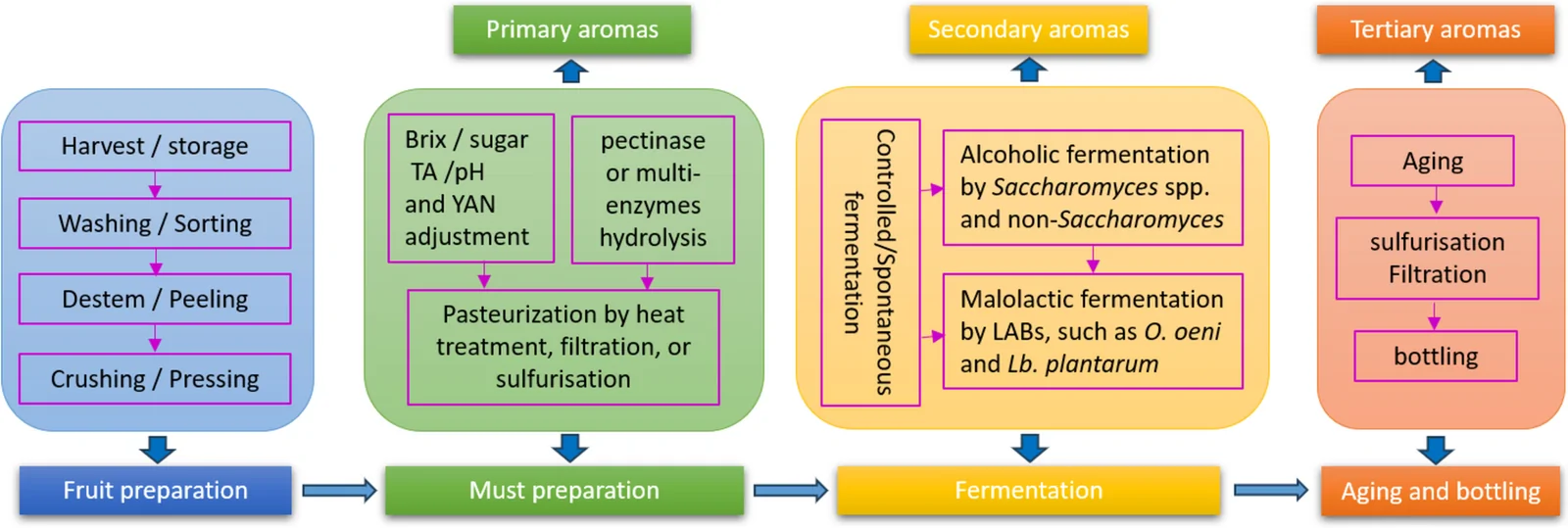
The fruit wine making process and the stages of aroma formation

Although fruit wines with different features provide many more choices for consumers’ demand for diverse wine varieties, the production process of all fruit wines follows a similar manufacturing procedure as grape wines. In brief, the fruit winemaking process includes the following main steps (Fig. 1): fruit harvesting and acquisition; extraction and preparation of fruits musts via crushing, clarifying, and adjusting; fermentation; maturation; aging; and bottling (Velić et al. 2018b). However, due to the differences in the nature of raw materials, some steps during the winemaking process may require special treatment techniques, making them distinctly different from grape winemaking, which finally affects their volatile profiles and quality (Ruppert et al. 2021).

During fruit must preparation, slightly overripe fruits containing a higher sugar and lower acid are more suitable for wine fermentation, resulting in the formation of an appropriate volatile aroma profile. However, an addition of sucrose and/or citric acid in the must is practiced for their suitable adjustment for winemaking (Lin et al. 2018). On the other hand, when it is difficult to obtain fresh fruit juice, concentrated juice is used for winemaking; under such conditions, the higher sugar content is adjusted by adding water, while the higher acidity is adjusted by adding calcium carbonate (Schelezki et al. 2018; Babincev & Jović 2023). Wang et al. (2018) reported that pH adjustment affected the formation patterns of esters, higher alcohols, and acids among the 93 volatiles in bog bilberry syrup wine, which altered its aroma profile during bottle-aging. Additionally, in case of the fruits containing higher pectin than grapes, the addition of pectinase or multi-enzyme preparations to degrade the pectin and other polysaccharides in fruit juice is required and conducive to increase juice yields, shorter time of maceration, settling, and filtration, which improves sugar release and color extraction; enhances volatile formation; and improves aroma/flavor profiles (Jagtap and Bapat 2015; Claus and Mojsov 2018). The application of commercial pectinases in juice and fruit wine manufacturing dates back to the 1930s (Kashyap et al. 2001). Almost all fruit wine making processes involve the addition of pectinases, usually in the form of free enzymes added to fruit pulps, while few in the form of immobilized enzymes (Martín et al. 2019). The amount of enzyme preparations added and catalytic temperature may vary depending on the type of fruits and pectinase itself (Table 1). Recently, a few studies were conducted to examine the impact of pectinase on the oenological parameters and aroma profiles of fruit wine. Aneh et al. (2023) demonstrated that pectinase treatment markedly improved the juice yield and total soluble solids of the atom fruit (*Dacryodes macrophylla*) wine but decreased vitamin C content compared to the non-pectinase-treated juice samples. The significantly increased yield of juice/wine was confirmed when red dragon pulps were pre-treated with pectinases followed by the fermentation with *Torulaspora delbrueckii* (Jiang et al. 2020). Moreover, higher levels of esters, terpenes, and total phenolic content but lower content of higher alcohols were detected in pectinase-treated samples, improving the aroma/flavor profiles of the red dragon wine. On the other hand, pectinase treatment also leads to a decrease in betacyanin content and color intensity, which may have an impact on the appearance of fruit wine (Jiang et al. 2020The next stage is the pasteurization of fruit juice, either through heat treatment, filtration, or by adding potassium metabisulfite to inhibit the growth of undesirable microorganisms during fermentation (Lisanti et al. 2019; Sumby et al. 2019). Unlike grape musts, many fruit juices lack nitrogenous nutrients, which often need the addition of yeast extract or diammonium phosphate (DAP) to maintain the growth of yeast, which has an impact on aroma profiles and the overall wine quality (Vilanova et al. 2015; Mendes-Ferreira et al. 2019; Xu et al. 2022a, b; Bell and Henschke 2005). The addition of 60 mg N/L DAP showed a significant increase in volatile compounds, especially fatty acids (medium chain) and their ethyl esters that contributed mostly to the tropical fruity aroma, whereas higher alcohols and acetate esters were remarkably increased with the supplementation of 150 mg N/L amino acids (Xu et al. 2022a, b). At fermentation stage, temperature always influences yeast growth, thereby impacting the volatile contents and aroma profile. Fermentation temperature is usually controlled between 4 and 16 °C or at least below 26 °C, leading to slower fermentation and the formation of a more harmonious aroma profile with more volatile components and alcohol retention (Matei 2017). In comparison with fermentation at 15 °C, fermentation at 25 °C consumed more sugars, significantly decreasing pH, and producing 30 volatile compounds in pomegranate wine; these volatile compounds included alcohols, organic acids, esters, and terpenes. Fermentation with *Saccharomyces cerevisiae* WB06 at 15 °C achieved a higher content of esters than alcohols, as confirmed by the principal component analysis (Kokkinomagoulos et al. 2020). Malolactic fermentation (MLF) was reported to benefit the flavor profile and mouthfeel of fruit wine by decreasing the acidity via the transformation of acid from malic to lactic, ultimately balancing volatile compound contents (Navarrete-Bolaños et al. 2013). During wine aging, to preserve the original unique flavors and aroma profiles, the use of wooden barrels is not necessary; however, during the aging of distilled beverages (such as fruit brandies), the traditional wooden barrel aging shows a marked impact on their final taste and aroma (Canas et al. 2016). Considering the cost-effectiveness and environmental sustainability, the use of chips, sticks, or even powders instead of oak barrels to enhance their sensory properties, flavor and phenolic profiles, and finally to facilitate the rapid aging of apple ciders (Fan et al. 2006) and fruit brandies (Nie et al. 2023; Sánchez-Guillén et al. 2019) is currently a feasible alternative.

**Table 1:** Commercial pectinases used in fruit wine making

Brand name	Supplier and location	Working concentration	Temperature and time	Fruit wine	References
Lallzyme EX-V pectinase	Scott Laboratories Petaluma, CA, USA	20 mg/L	20 °C, 1 h	Black raspberry wine, cherry wine, raspberry wine, and mulberry wine	Liu et al. (2020b), Sun et al. (2016b), Li et al. (2020), and Zhang et al. (2022b)
Pectinex® Ultra SP-L	Novozymes, Demark	0.1% (v/v)	40 °C, 1 h	Red dragon fruit wine	Jiang et al. (2020)
		0.25% (v/v)	25 °C, 20 min	Strawberry wine	Yang et al. (2021)
Lallzyme HC™	Lallema, Canada	20 mg/L	/	Apple wine	Bandić et al. (2019)
Pectinase	Beijing Solarbio Science & Technology Co. Ltd., Beijing, China	1% (v/v)	40 °C, 2 h	Longan fruit wine	Liu et al. (2018)
Pectinase	/	20 mg/L	37 °C, 1 h	Kiwi wine	Li et al. (2022a, b)
Pectinase	/	0.5 g/L	45 °C, 2 h	Peach wine	Liu et al. (2020a)
Pectinase	/	0.1% (v/v)	20 °C, 10 h	Lemon wine	Wu et al. (2023)
Pectinase	/	40 mg/L	40 °C, 10 h	Orange wine	Liu et al. (2023)
Pectinase	/	50 mg/kg	40 °C, 2 h	Blueberry wine	Wang et al. (2022)

*Note*: The forward slash (/) indicates that no information about the supplier was mentioned in the references

## Raw materials for fruit wine making

In general, fruits are rich in nutritional components (carbohydrates, amino acids, vitamins, minerals, dietary fibers, etc.) as well as bioactive phytochemicals (polyphenolic compounds, flavonoids, etc.) (Kumar and Singh 2021). The nutrients and contents may vary from the fruit species, varieties, and cultivars. Meanwhile, the fruit maturity, cultivation mode, plant region, and climate may have impacts on the nutrient compositions and fruit quality, ultimately affecting the quality of wine and its volatile profiles (El Hadi et al. 2013).

Variations in the concentration of fructose, sucrose, and glucose as well as citric, malic, and succinic acids in fruits significantly affect both the alcoholic and malolactic fermentation, leading to effects on volatile profiles and fruit wine quality. Sugars are fermented mainly by yeast during AF, producing ethanol, fatty acid esters, glycerol, aldehydes, and carbon dioxide. Ethanol contributes to wine-like odor as well as serves as a co-solvent along with water to extract organic compounds with various aroma notes and/or colors from fermented fruit musts, which react with other compounds to synthesize ethyl esters like important volatile compounds, contributing to the sensory profile and stability of fruit wine (Charoenchai 2019). Glycerol contributes to the sweetness, the full and round mouthfeel of wine, and wine texture (Laguna et al. 2017). As metabolic intermediates, organic acids are not only metabolites of organic compounds (for example, sugars and lipids) but also can be used as substrates for fermentation to generate volatile aromatic compounds like esters, alcohols, and aldehydes during fermentation (Vicente et al. 2022). The organic acids (mainly including malic acid, citric acid, and acetic acid) contribute not only dominant flavor of sourness but also bitterness and astringency (Ju et al. 2023). The fruit acidity affects the acidity and the taste of fruit wine. Acetic acid is one of the predominant volatile acids, which, if present in higher concentrations, negatively affect the organoleptic properties of fruit wines (Vilela-Moura et al. 2010, 2011). Malic acid also significantly contributes to the acidity of the wine, which can be transformed into lactic acid by MLF with an increase in pH and a decrease in total acidity (Vicente et al. 2022).

Nitrogenous compounds in fruits and musts in inorganic forms (ammonium salt) and organic forms (amino acids) serve as nitrogen sources for yeast multiplication during fermentation and can transform a broad number of wine compounds, which, in turn, potentially affect volatile profiles and wine quality (Sharma et al. 2017). Amino acids, as the primary precursors of many volatile compounds, can be converted into alcohols, ketones, aldehydes, acids, and esters through deamination, transamination, or decarboxylation by wine microbes or fruit endogenous enzymes during fermentation. Lee et al. (2011) found in papaya wine that the aroma profile was affected by the inclusion of selected amino acids (leucine, isoleucine, valine, and phenylalanine). The addition of glutathione can increase the content of higher alcohols (2-methyl-1-propanol, 1-heptanol, 3-methyl-1-butanol, and phenylethyl alcohol), possibly synthesizing via Ehrlich pathway (Xu et al. 2019) and the content of terpene alcohols (geraniol, citronellol, and linalool), imparting floral aroma (Cai et al. 2014). Moreover, such addition of glutathione in Fuji apple wine increases the contents of n-butyl acetate, ethyl benzoate, and ethyl 9-decenoate, contributing to fruity fragrance of the wine, while it decreases the contents of 2-methyl butyric acid, acetic acid, and acetaldehyde, contributing the complex fragrance of the wine at a lower concentration; however, their concentration above threshold values impact negatively with a pungent aroma (Cai et al. 2014; Xu et al. 2019).

Phenolic compounds are secondary plant metabolites that mainly originated from fruits and partially formed or degraded during fermentation. Thus, the phenolic contents of fruit wines depend on the portion of fruits, fruit types, fruit species, variety or cultivar, plant location, climate, harvest year, maturity, storage, and processing (Liu et al. 2020a, b; Bandić et al. 2019; Zhao et al. 2023; van der Sluis et al. 2001; Zhang et al. 2021a; Li et al. 2022a, b). Fruit wines made from cherry, raspberry, bilberry, elderberry, blueberry, black currant, and sea buckthorn contain a comparable or higher total phenolic compounds than that of grape red wines, while the fruit wines made from apple, pear, strawberry, plum, and peach have a lower level of such compounds (Rupasinghe and Clegg 2007; Dey et al. 2009). Ethyl phenols are the representatives of volatile phenols produced by some strains of LAB and yeasts within the genus *Brettanomyces* (Madsen et al. 2017), which give unpleasant horse sweat, leather, and stable odors. The preferential selection and employment of non-*Saccharomyces* yeast, including *Torulaspora delbrueckii* (Wei et al. 2020) and *Wickerhamomyces anomalus* (Li et al. 2022a, b), as well as lactic acid bacteria, including *Oenococcus oeni* and *Lactobacillus plantarum* (Devi et al. 2020), effectively alter the content of anthocyanins and other phenolic compounds by metabolic activities of wine microbes (Costello et al. 2012) or enzymatic reactions (β-glucosidase and pectinase) (Minnaar et al. 2019; Choi et al. 2020) during fruit wine fermentation, affecting the product sensory characteristics.

## Impact of wine fermenting microbes on volatile profiles and wine quality

Fermentation microorganisms (mainly yeasts and lactic acid bacteria) are the main executors of fruit wine fermentation and predominant producers of aroma compounds, playing a crucial role in determining the quality of fruit wine. The *Saccharomyces*, non-*Saccharomyces*, and LAB strains commonly used in fruit winemaking in recent years were summarized and listed in Table 2.

**Table 2:** Yeast and lactic acid bacteria strains for fruit wine fermentation

Fruit	Yeasts or lactic acid bacteria	Ref
Apple	*Saccharomyces bayanus* Fermol Blanc, *Saccharomyces cerevisiae* EC 1118; *S. cerevisiae* CCTCC M201022; *Saccharomyces uvarum*, *Torulaspora delbrueckii* TD291, *Hanseniaspora osmophila*, *H. uvarum*, *Starmerella bacillaris*, *Zygosaccharomyces bailii*; *S. cerevisiae* var. *bayanus*; *H. osmophila* X25-5, *T. quercuum* X24-4; *S. cerevisiae* WLP775, *S. cerevisiae* WLS21; *Wickerhamomyces anomalus* YN6	Bandić et al. (2019), Fan et al. (2006), Lorenzini et al. (2019), Ruppert et al. (2021), Wei et al (2020), Xu et al. (2022a, b), and Ye et al. (2014b)
Apricot	*Pichia kudriavzevii* LQD20, *P. kudriavzevii* LQD5; *S. cerevisiae* var. *bayanus* EC-1118	Chen et al. (2023) and Choi et al. (2020)
Bilberry	*S. cerevisiae* 1116, *T. delbrueckii* 291, *T. delbrueckii* 70526, *Schizosaccharomyces pombe* 3796, *S. pombe* 70572	Liu et al. (2019a, b)
Blackberry	*S. cerevisiae* ICV K1, *S. cerevisiae* Enoferm T306, *S. cerevisiae* Vitilevure CM4457, *S. cerevisiae* Greroche Rhona L3574	Bautista-Rosales et al. (2014)
Black raspberry	*S. cerevisiae* Lalvin RHST, *T. delbrueckii* Viniflora Prelude™, *Oenococcus oeni* Viniflora Oenos	Liu et al. (2020b)
Blueberry	*S. cerevisiae* Actiflore F33, Lalvin QA23, *T. delbrueckii* Zymaflore ® Alpha	Wang et al. (2022)
Cherry	*S. cerevisiae* Lalvin RC212, *T. delbrueckii* 49; *S. cerevisiae* Lalvin GRE, *O. oeni* 31MBR, *Lb. plantarum* SGJ-24	Sun et al. (2016a) and Sun et al. (2016b)
Fig	*S. cerevisiae* RV002, RV171, BV818, PHYT, LM-8, and FM-sc-08	Ma et al. (2022)
Hawthorn	*S. cerevisiae* EC1118, SYRAH, M2, MT, 2226, RA17, KD, D47, DV10, CSM, 2323, UV43, D80, Clos, FC9, 71B, OKAY, IOAYS, QA23, BDX, CM, SIHA Active 3, SIHA Active 7	Han et al. (2021)
Kiwifruit	*W. anomala* BLCC12 (Wa), *Z. rouxii* IFO30, *Z. bailii* IFO37, *S. pombe* 1757, *S. cerevisiae* WLS21	Li et al. (2022b)
Lemons	*S. cerevisiae* RW, *S. cerevisiae* DV10, *S. cerevisiae* 71B	Wu et al. (2023)
Litchi	*S. cerevisiae* R2, EC1118, EC1119, MERIT.ferm, and 71	Chen et al. (2016)
Longan	*S. cerevisiae* F33; Lalvin71B, Lalvin EC1118, Lalvin D254, Lalvin RC212, Lalvin RC2323, Lalvin K1, Lalvin U43, Lalvin KD, and Lalvin R-HST; Angel BV818, Angel SY, and Angel RW	Liu et al. (2018)
Mango	*Pichia kudriavzevii* HDX1, *P. kudriavzevii* HDX1, *P. kudriavzevii* HDY1, and *P. kudriavzevii* HDA2	Bao et al. (2021)
Mangosteen	*S. cerevisiae* GRE, Lalvin RC212, Lalvin D254, CGMCC2.23, CGMCC2.4	Xiao et al. (2015)
Mulberry	*S. cerevisiae* GRE, *O. oeni* of Viniflora ® CH11, *Lb. plantarum* of Bactoferm ® vege-start 2.0 CN; *S. cerevisiae* Ruby	Zhang et al. (2022b)
Orange	*S. cerevisiae* Sc NCUF309.2, *T. delbrueckii* Td NCUF305.2	Liu et al. (2023)
Passion fruit	*S. cerevisiae* ES488 and CY3079, *S. bayanus* BV818 and VIC	Liu et al. (2022a)
Peaches	*S. cerevisiae* PY01, WT-21, *H. uvarum* #15, *M. pulcherrima* #30, *Lachancea thermotolerans*, and *T. delbrueckii* Zymaflore® AlphaTD N. SACCH	Liu et al. (2020a) and Liu et al. (2022b)
*Rosa roxburghii*	*S. cerevisiae* ZYMAFLORE X16, *H. uvarum* 32,349, *H. uvarum* F119	Huang et al. (2022)
Persimmon	*S. cerevisiae* BV818*, S. cerevisiae* var. *ellipsoideus* UCD 595	Nie et al. (2023) and Sharma et al. (2017)
Pineapples	*S. cerevisiae* D254, VIC, BV818, and CECA	Lin et al. (2018)
Plum	*S. cerevisiae* var. *burgundy*, *H. thailandic*a Zal1, *S. cerevisiae* Lalvin EC1118	Nanthavut et al. (2023)
Pomegranates	*S. bayanus* SN9, *S. cerevisiae* M02-Cider, *S. cerevisiae* var. *diastaticus* SAFALE™ WB-06	Kokkinomagoulos et al. (2020)
Ponkan	*S. cerevisiae* BCRC 21761, BCRC 21805, BCRC 21823, BCRC 22293, BCRC 22332, and HF-08	Lee et al. (2013)
Prickly pear	*S. cerevisiae*, *P. fermentans*, *O. oeni*	Navarrete-Bolanos et al. (2013)
Raspberry	*S. cerevisiae* L’ Authentiqu, *T. delbrueckii* Biodiva	Li et al. (2020)
Strawberries	*S. cerevisiae* Lalvin V1116, *T. delbrueckii* Biodiva	Yang et al. (2021)
Tangerines	*S. cerevisiae* (Angel yeast)	Xu et al. (2022a, b)
Wax apple	*S. cerevisiae* D254, VIC, BV818, and RV100	Lin et al. (2019)

Yeasts are the main producers of alcohol during fruit wine fermentation, which plays a critical role in volatile production, imparting aroma quality. In addition, they also produce higher alcohols, fatty acids, and esters, imparting to the flavor profile. *S. cerevisiae* generally produces higher concentrations of acetaldehydes and esters but lower concentrations of higher alcohols (Capozzi et al. 2015). Pentanoic, hexanoic, octanoic, decanoic, and 9-decenoic acid like aliphatic fatty acids are synthesized by *S. cerevisiae* and acetic acid and isobutyric acid mainly produced by non-*Saccharomyces* species like *Hanseniaspora* spp. and *Candida* spp. can be converted to corresponding ethyl ester, which significantly contributes to the fruity aroma but they also impart rancid, greasy, and cheesy notes at excessive concentration (Jeromel et al. 2019; Lorenzini et al. 2019). The Ehrlich pathway generates 1-propanol, isobutanol, and isoamyl alcohol like higher alcohols (Xu et al. 2019), which may get involved in the synthesis of aroma compounds as ester precursors (Styger et al. 2011). Other volatile compounds, including 2-methylbutanol-1, 2-phenylethanol, 3-methyl-1-butanol, or isoamyl alcohol usually present in fruit wine (Ye et al. 2014a, b), have a positive influence on the aroma profile of fruit wine at an appropriate concentration by providing flower, honey, and fruit notes (Jeromel et al. 2019; Perestrelo et al. 2020). Esters are a large group of aroma compounds, holding different classifications, such as acetate esters and ethyl fatty acid esters; they contribute flowery, fruity, and honey notes to the wine aroma profile. Alcohol and higher alcohols are responsible for the formation of these esters (Jeromel et al. 2019; Perestrelo et al. 2020); ethyl fatty acid esters comprise ethyl octanoate, ethyl hexanoate, and ethyl decanoate, while acetate esters comprise different acetates (isobutyl-, amyl-, hexyl-, ethyl-, isoamyl-, and 2-phenylethyl-acetate). However, excessive amounts of esters may provide unpleasant flavor to the aroma profile and quality of the wine.

Yeast species, mainly *S. cerevisiae*, act as the main microbes for alcoholic fermentation (AF), playing an essential role in converting sugars into alcohol and various volatile compounds and contributing to producing sensory features during fruit winemaking (Takush and Osborne 2012). Different strains of *Saccharomyces*, even the commercial strains, may show respective fermentation capabilities, affecting the volatile compositions of fruit wines fermented from diverse fruit juices (Xiao et al. 2015). Among the effects of four strains of *S. cerevisiae* (VIC, RV100, D254, and BV818), the volatile content of wax apple wine with the strain VIC was the highest, giving the wine the intense aroma because of the highest number and content of volatile compounds. The other two strains (RV100 and D254) formed the highest volatile varieties with the highest score in overall flavor, enhancing the flavor complexity (Lin et al. 2019). Sensory evaluation in another study confirmed the pronounced influence of the yeasts on wine quality and singled out the commercial strain *S. bayanus* Fermol Blanc which was found to be a better starter for apple wine (Cripps Pink and Idared variety) in comparison with the strain EC 1118 (Bandić et al. 2019).

Non-*Saccharomyces*, which usually multiply at the beginning of the AF stage followed by the suppression of *S. cerevisiae*, have been demonstrated to increase yields of desirable volatile compounds, contribute to aroma complexity, and finally modulate organoleptic property through interaction with *S. cerevisiae* (González et al. 2018; Jolly et al. 2014). Among the non-*Saccharomyces* strains, *Candida zemplinina* (Medina, et al. 2013), *Schizosaccharomyces pombe* (Benito et al. 2016), *Hanseniaspora vineae* (Englezos et al. 2018), and *T. delbrueckii* (Li et al. 2020; Sun et al. 2016a) are the best species for the production of ethanol during (Benito 2018). Notably, *Pichia kluyveri* and *T. delbrueckii* like yeasts are commercially available as active dry or active frozen forms (Chasseriaud et al. 2018). Studies reported that inoculation with *T. delbrueckii* enhanced the complexity of wine aroma; however, it decreased the content of acetic acid like volatile acid (Benito 2018; Liu et al. 2019a). Co-fermentation with *S. cerevisiae* and *T. delbrueckii* is possibly a suitable strategy for wife fermentation from bilberry and raspberry (Liu et al. 2019a; Li et al. 2020). Other strains of non-*Saccharomyces* species, such as *Metschnikowia pulcherrima*, *H. osmophila*, *H. uvarum*, *Starmerella bacillaris*, *Zygosaccharomyces bailii*, *Williopsis saturnus*, *Kloeckera apicule*, *Pichia anomala*, and *P. kluyveri*, have also been proven to diversify aroma and are beneficial to flavor profiles of wine (Benito et al. 2016; Zhang et al. 2022a; Escribano-Viana et al. 2018; Li et al. 2022a, b; Lorenzini et al. 2019; Lee et al. 2011; Jolly et al. 2006). One of the main reasons that non-*Saccharomyces* species can improve the flavor profiles of fruit wines is that they can produce a variety of higher activity β-glucosidases than *Saccharomyces* yeasts (Zhang et al. 2021b). The β-glucosidase breaks the glycosidic bonds of glycoconjugates, which exist as flavorless and non-volatile forms (Maicas and Mateo 2005) in juices/wines of various fruits such as apples, blueberries, plums, and strawberries, releasing volatile terpenes (such as germaniol, nerol, citronellol, linalool, and α-terpineol) (Liu et al. 2019b), phenylprops (e.g., eugenol) (Atkinson 2018), norisoprenoids (such as α-ionone, β-ionone, β-cyclocitral, and β-damascenone) (Mahattanatawee et al. 2005), and specific aliphatic esters during wine fermentation and eventually enhancing aroma/flavor profiles of fruit wine (Liang et al. 2020). In summary, the fruit wine fermented by non-*Saccharomyces* yeasts was found to have distinctly different aroma profiles and quality features, depending on the yeast species, the inoculation styles (co-inoculation or sequential inoculation), the fruit variety or cultivar, and nutritional compositions of the fruit. Therefore, more detailed and comprehensive research should be discreetly considered and encouraged.

Lactic acid bacteria (LAB), either via a spontaneous way by indigenous LAB or via a restrained way by selected inoculation of starter cultures, form lactic acid and carbon dioxide using malic acid (Ribéreau-Gayon et al. 2006). They modulate wine flavor compounds by many different enzymatic reactions of metabolic pathways, leading to decrease in acidity, increase in microbial stability, and contributing (positively or negatively) to aroma profile, including astringency, sweetness, bitterness, and acidity (Betteridge et al. 2015; Sun et al. 2016b). Diacetyl, which is an essential aromatic compound produced from citrate fermentation by LAB during MLF (Bartowsky and Borneman 2011), shows a lower odor threshold of the typical buttery, nutty aromas of wine (Bartowsky and Henschke 2004). It can be reduced to 2,3-butanediol and acetoin (both of them holds a higher threshold for odor), thus dramatically affecting the wine aroma (Virdis et al. 2020). LAB can release sugar-bound primary aromatic compounds, such as volatile phenols, C13 norisoprenoids, terpenes, and C6 compounds (Baumes 2009), which are odorless in their bound through glycosidases (Fia et al. 2018; Iorizzo et al. 2016). LAB can also liberate 3-sulfanylhexyl acetate and 3-sulfanylhexan-1-ol like volatile thiols, which are a group of important aromatic compounds with grapefruit and passionfruit notes from their non-odorant precursors through enzymes not characterized (Takase et al. 2018). LAB can alter the fruit wine ester profile according to the strain used (Gammacurta et al. 2018) and depending on the inoculation strategy (Lasik-Kurdyś et al. 2018). It is likely possible that the co-inoculation enhances the release of ethyl lactate, ethyl acetate, and diethyl succinate like ethyl esters, which enrich the fruity and floral aroma of the wine (concentration dependent) (Lasik-Kurdyś et al. 2018). Besides, LAB has also been reported to produce volatile phenols with unpleasant odors (Silva et al. 2011) and acetaldehyde with the influence of aroma, microbiological stability, and color (Liu and Pilone 2000).

Currently, the LAB strains used in MLF are usually *Lactobacillu*s, *Pediococcus*, *Oenococcus*, *Weissella*, and *Leuconostoc* spp. (Krieger-Weber et al. 2020; Virdis et al. 2020). Among them, both *O. oeni* and *Lb. plantarum* are the species, which are the starter culture for traditional MLF because they can tolerate harsh fermentation condition, such as excessive ethanol, high acidity, and low nutrients (Ribéreau-Gayon et al. 2006), which contribute to the sensory profile of wines (Chen and Liu 2016; Liu et al. 2020b; Ji et al. 2023). Chen and Liu (2016) reported that MLF enhanced the formation of aroma compounds, such as geraniol, isoamyl acetate, cis-rose oxide, and linalool, suggesting that MLF conducted by *O. oeni* retains the lychee flavor effectively. Sequential fermentation with *T. delbrueckii*, and *S. cerevisiae*, followed by the controlled MLF with *O. oeni* exhibited strong ‘fruity’ and light ‘solvent’ plus ‘herbaceous’ flavor, respectively, by producing significantly higher concentrations of esters (fruity flavor) and lower concentrations of alcohols (solvent flavor). This indicates that controlled MLF with *O. oeni* can affect positively on the flavor profiles of black raspberry wine (Liu et al. 2020b). Besides, *Lb. plantarum* exhibits a more diverse enzymatic profile than *O. oeni*, possibly leading to aroma profile modification (Brizuela et al.^2019^). The application of *Lb. plantarum* strains SGJ-24 showed a greater fermentation efficiency and produced more volatile esters and terpenes than that of the commercial *O. oeni* 31MBR, suggesting that *Lb. plantarum* SGJ-24 is a worthwhile alternative species for cherry wine (Sun et al. 2016b). Moreover, the use of mixed MLF starters for mulberry wine fermentation produced a better aroma profile than a single MLF. A mixed MLF using *Lb. plantarum* and *O. oeni* at a ratio of 4:1 scored highest in overall aroma; this is mainly based on the increased fruity and decreased ‘pungent’ odors in wine (Zhang et al. 2022b). Thus, it is concluded that the mixed MLF strategy is reasonable and practical to improve the sensory properties of fruit wines.

## Concluding remarks

Fruit wines are non-distilled alcoholic beverages, made by fermenting various non-grape fruits. The fruit varieties, growing areas and climates, harvesting seasons, and fruit nutritional contents all decide the flavor quality of the wine produced. Moreover, the selection of fermenting organisms and their fermentation conditions are the crucial and determining factors for wine sensory profiles owing to their different volatile production during fermentation. Therefore, we have noticed and reviewed the latest developments in this area that differ from grape wine making, and we agree with other peers that a wise selection of fruits; fermentation microorganisms, especially *Saccharomyces* species and LAB strains; and the appropriate fermentation conditions are prerequisites for a fruit wine having the desired flavor profiles and quality characteristics. In addition, the studies on the diversity and dynamics of microflora in fruit winemaking should be emphasized.

## Supplementary Information

Below is the link to the electronic supplementary material.

Supplementary file1 (PDF 259 KB)

## Data Availability

Data will be made available on reasonable request.
