# Supplementary material for: The formation of volatiles in fruit wine process and its impact on wine quality
Source: Appl Microbiol Biotechnol. 2024 Jul 17;108:420. doi: 10.1007/s00253-024-13084-8 (PMC11254978; doi:10.1007/s00253-024-13084-8)
Supplement: Supplementary file 1 [file 253_2024_13084_MOESM1_ESM.pdf]

Journal name: Applied Microbiology and Biotechnology

The formation of volatiles in fruit wine process and its impact on wine quality

Jianxin Tan*[#], Mingyue Ji[#], Jiangang Gong, Bimal Chitrakar*

College of Food Science and Technology, Hebei Agricultural University, Baoding 071001, People's Republic of China

* Corresponding author: Jianxin Tan (jianxintan@sina.com) and Bimal Chitrakar (bimal@hebau.edu.cn), Phone number: 86-312-7528180, Fax: 86-312-7528180

Table S1 Fruits and their volatile compounds in fruit wines

Fruits	Alcohols	Acetate esters	Ethyl esters	Other esters
Apple (*Malus domestica* Borkh.)	(6) 1-Butanol, 2-Butanol, 3-Methyl-1-butanol, 2-Phenylethyl alcohol, 1-Hexanol, Benzyl alcohol	(2) Hexyl acetate, Isoamyl acetate	(7) Ethyl butyrate, Ethyl isovalerate, Ethyl hexanoate, Ethyl octanoate, Ethyl decanoate, Ethyl lactate, Ethyl cinnamate	
Apple (*Malus domestica* Borkh.)	(14) 2-Methyl-1-butanol, 3-Methyl-1-butanol, 1-Pentanol, 2,3-Butanediol, 3-Ethoxy-1-propanol, 3-Hexen-1-ol (*E*), 1-Hexanol, 1-Heptanol, 2-Methyl-6-hepten-1-ol, 3-Ethyl-4-methylpentan-1-ol, 2-Ethylhexanol, Benzyl alcohol, 1-Octanol, Phenylethyl alcohol	(8) Ethyl acetate, Propyl acetate, Butyl acetate, 2-Methylbutyl acetate, 3-Methylbutyl acetate, Hexyl acetate, Ethylphenyl acetate, Phenethyl acetate	(12) Ethyl butanoate, Ethyl propanoate, Ethyl hexanoate, Ethyl benzoate, Ethyl octanoate, Ethyl decanoate, Ethyl dodecanoate, Ethyl tetradecanoate, Ethyl-9-hexadecenoate, Ethyl hexadecanoate, Ethyl 2-methyl butanoate, Ethyl-3-hydroxy butanoate	(14) Methyl hexanoate, Methyl octanoate, Methyl decanoate, Methyl hexadecanoate, Methyl dodecanoate, 2-Methylpropyl octanoate, 3-Methylbutyl octanoate, 3-Methylbutyl decanoate, 2-Methylbutyl decanoate, 3-Methylbutyl dodecanoate, 2-Phenylethyl hexanoate, Hexyl octanoate, Isoamyl lactate
Apricot (*Prunus armeniaca* L.)	(10) 1-Propanol alcohol, Isobutanol alcohol, Isoamyl alcohol, 1-Hexanol, 3-Ethoxypropanol, 1-Octanol, 2,3-Butanediol, 1-Decanol, Benzyl alcohol, Phenylethyl alcohol	(9) Methyl acetate, Ethyl acetate, Geranyl acetate, Propyl acetate, Isobutyl acetate, Butyl acetate, Isoamyl acetate, Hexyl acetate, 2-Phenylethyl acetate	(13) Ethyl propionate, Ethyl isobutyrate, Ethyl octanoate, Ethyl butanoate, Ethyl nonanoate, Ethyl decanoate, Ethyl benzoate, Ethyl 9-decenoate, Ethyl pentanoate, Ethyl hexanoate, Ethyl	(3) Methyl octanoate, Methyl decanoate, Methyl salicylate

			heptanoate, Ethyl phenylacetate, Ethyl dodecanoate	
Bilberry (*Vaccinium myrtillus* L.)	(16) 1-Hexanol, (*Z*)-3-Hexen-1-ol, (*E*)-2-Hexen-1-ol, 1-Propanol, 2-Methyl-1-propanol, 1-Butanol, 2-Methyl-1-butanol, 3-Methyl-1-butanol, 4-Methyl-1-pentanol, 3-Methyl-1-pentanol, 3-Ethoxy-1-propanol, 1-Heptanol, 2-Ethyl-1-hexanol, 1-Octanol, Threo-2,3-butanediol, 2-Phenylethanol	(4) Ethyl acetate, 4-Methyl-2-pentyl acetate, Isoamyl acetate, Phenethyl acetate	(9) Ethyl 3-methylbutyrate, Ethyl hexanoate, Ethyl heptanoate, Ethyl lactate, Ethyl caprylate, Ethyl caprate, Ethyl 9-decenoate, Ethyl 4-hydroxybutanoate, Ethyl dodecanoate	(5) Methyl 2-hydroxy-3-methylbutanoate, Isopentyl 3-methylbutyrate, 2,6,8-Trimethyl-4-nonanol, Methyl decanoate, Diethyl succinate,
Black raspberry (*Rubus occidentalis* L.)	(8) Ethanol, Isopropyl alcohol, 1-Propano, 2-Methyl-1-propanol, 1-Butanol, 3-Methyl-3-buten-1-ol, 3-Methyl-1-pentanol, 1-Hexanol	(4) Ethyl acetate, Propyl acetate, Isobutyl acetate, Hexyl acetate	(12) Ethyl formate, Ethyl propanoate, Ethyl isobutyrate, Ethyl lactate, Ethyl butyrate, Ethyl 2-methylbutanoate, Ethyl isovalerate, Ethyl hexanoate, Ethyl heptanoate, Ethyl 2-hexenoate, Ethyl octanoate, Ethyl nonanoate	(2) Methyl 2-methylbutanoate, Isoamyl acetate
Blueberry (*Ericaeae Vaccinium*)	(11) P-cymene-8-ol, 3-Methyl-1-pentanol, 4-Methyl-1-pentanol, 1-Octanol, Isoamyl alcohol, 1-Decanol, 2-Nonanol, 1-Dodecanol, 1-Nonanol, 1-Octen-3-ol, Isobutanol	(6) Ethyl acetate, Isobutyl acetate, Isoamyl acetate, 2-methylbutyl acetate, β-phenethyl acetate, Linalyl acetate	(10) Ethyl decanoate, Ethyl octanoate, Ethyl 2-hexenoate, Ethyl hexanoate, Ethyl linoleate, Ethyl hexadecanoate, Ethyl butanoate, Ethyl 3-methylbutanoate, Ethyl phenylacetate, Ethyl isopentyl succinate	(4) Methyl octanoate, Isoamyl formate, Methyl cinnamate, Methyl isovalerate
Blueberry (*Vaccinium corymbosum* L.)	(13) Methanol, 1-Propanol, 2-Methyl-1-propanol, 2-Methyl-1-butanol, 3-Methyl-1-butanol, 2,3-Butanediol, 2,3-Butanediol, 2-Phenylethanol, *Z*-3-Hexenol, 3-Methyl-1-pentanol, 1-Hexanol, 3-Ethoxy-1-propanol, Benzyl alcohol	(2) Ethyl acetate, Isoamyl acetate	(4) Ethyl lactate, Ethyl butyrate, Ethyl hexanoate, Ethyl octanoate	(5) Methyl vanillate, Monoethyl succinate, Diethyl malate, Diethyl succinate, Diethyl malonate

Kiwi fruit (*Actinidia chinensis*)	(13) 2-Methyl-1-propanol, 1-Butanol, 3-Methyl-1-butanol, 2-Methyl-1-butanol, (*R*, *R*)-2,3-butanediol, (+-)-5-Methyl-2-hexanol, 1-Hexanol, Cis-3-hexen-1-ol, Trans-3-hexen-1-ol, Trans-2-hexen-1-ol, 1-Heptanol, 1-Octanol, 2-Phenyl-1-ethanol	(11) Methyl acetate, Ethyl acetate, Propyl acetate, Isobutyl acetate, Butyl acetate, Isoamyl acetate, 2-Methylbutyl acetate, Hexyl acetate, Heptyl acetate, Octyl acetate, Phenethyl acetate,	(14) Ethyl propanoate, Ethyl isobutyrate, Ethyl butyrate, Ethyl 2-methylbutyrate, Ethyl pentanoate, Ethyl hexanoate, Ethyl heptanoate, Ethyl benzoate, Ethyl octanoate, Ethyl nonanoate, Ethyl 9-decenoate, Ethyl decanoate, Ethyl undecanoate, Ethyl dodecanoate	(22) Methyl butyrate, Methyl pentanoate, Isobutyl isobutyrate, Methyl hexanoate, Propyl butyrate, Butyl isobutyrate, Isobutyl butyrate, Butyl butyrate, Isoamyl butyrate, Methyl benzoate, Methyl octanoate, Isobutyl hexanoate, Hexyl butyrate, Isopentyl hexanoate, Methyl decanoate, Methyl dec-4-enoate, Isobutyl octanoate, Isoamyl octanoate, Methyl dodecanoate, Isobutyl decanoate, Isoamyl decanoate, Decyl decanoate
Longan (*Dimocarpus longan* Lour.)	(12) 1-Butanol,1-Hexanol, Isoamyl alcohol, 1-Pentanol, (*S*)-2-Octanol, 2-Undecanol, Phenylethyl alcohol, 6-Methyl-5-hepten-2-ol, 2-Methyl-1-propanol, 3-Ethyl-1-butanol, 2-(2-Ethoxyethoxy)-ethanol, 3-Methyl-2-methylene-3-buten-1-ol	(8) Ethyl acetate, 2-Methylpropyl acetate, 3-Methylbutyl acetate, Hexyl acetate, Citronellyl acetate, 1-Octen-3-yl acetate, 2-Phenylethyl acetate, (2*E*,4*E*)-2,4-Hexadienyl acetate	(14) Ethyl butanoate, Ethyl hexanoate, Ethyl-2-hexenoate, Ethyl heptanoate, Ethyl octanoate, Ethyl nonanoate, Ethyl decanoate, Ethyl-9-decanoate, Ethyl dodecanoate, Ethyl pentadecanoate, Ethyl hexadecanoate, Ethyl sorbate, Ethyl 2-hydroxypropanoate, Ethyl benzoate	(10) Methyl decanoate, Methyl octanoate, Methyl salicylate, Methyl dodecanoate, Butylcaprylate, Isopentyl hexanoate, Isobutyl hexanoate, Isopropyl pentakis(trimethylsilyl) diorthosilicate, 2-Octyl-cyclopropane tetradecanoic acid methyl ester, 3,7-Dimethyl-6-octenoic acid methyl ester
Mango (*Mangifera indica* L.)	(8) Alcohol Ethanol, Isopentanol, 3-Methylthiopropanol, Phenethyl alcohol, 1-Pentanol, Heptanol, p-Mentha-1,5-dien-8-ol, n-Decanol,	(2) Ethyl acetate, Phenethyl acetate	(5) Ethyl propanoate, Ethyl octanoate, Ethyl myristate, Methyl phthalate, Ethyl palmitate	(5) Dihydroactinidiolide, Methyl butyrate, *n*-Octyl formate, *γ*-Octanoic lactone, Isoamyl phenylacetate

Mulberry (*Morus* spp.)	(10) Methanol, Ethanol, 1-Propanol, 2-Propanol, 1-Butanol, 2-Methyl-1-propanol, 3-Methyl-1-butanol, 3-(Methylthio)-1-propanol, 1-Hexanol, 1-Penten-3-ol	(6) Methyl acetate, Ethyl acetate, 2-Methylpropyl acetate, Propyl acetate, 2-Methylbutyl acetate, Isoamyl acetate	(10) Ethyl lactate, Ethyl propanoate, Ethyl octanoate, Ethyl butanoate, Ethyl isobutyrate, Ethyl 3-methylbutanoate, Ethyl 2-methylbutanoate, Ethyl hexanoate, Ethyl formate	
Orange (*Citrus sinensis* Osb. var. *brasiliensis* Tanaka)	(3) Isoamyl alcohol, 2-Methyl-1-butanol, Phenylethanol	(3) Isoamyl acetate, 2-Methylbutyl acetate, Phenethyl acetate	(5) Ethyl propionate, Ethyl hexanoate, Ethyl 3-hydroxyhexanoate, Ethyl benzoate, Ethyl octanoate	*n*-Hexyl formate
Passion fruit (*Passiflora edulis*)	(8) Isobutanol, Isopentanol, 2-Heptanol, 2-Nonanol, 1-Octanol, 1-Decanol, Phenyl ethyl alcohol, Benzyl alcohol	(6) Isobutyl acetate, Isopentyl acetate, Hexyl acetate, Octyl acetate. Benzyl acetate, Phenethyl acetate	(16) Ethyl acetate, Ethyl butanoate, Ethyl hexanoate, Ethyl 3-hexenoate, Ethyl heptanoate, Ethyl 2-hexenoate, Ethyl caprylate, Ethyl 3-hydroxybutyrate, Ethyl caprate, Ethyl 3-hydroxyhexanoate, Ethyl 9-decenoate, Ethyl laurate, Ethyl tetradecanoate, Ethyl palmitate, Ethyl 9-hexadecenoate, Ethyl cinnamate	(10) Isobutyl hexanoate, Methyl octanoate, Hexyl butyrate, Isopentyl hexanoate, 2-Heptyl hexanoate, Methyl decanoate, isoamyl caprylate, Diethyl succinate, Isoamyl decanoate, Methyl salicylate
Peach (*Prunus persica* L.)	(7) 2-Methyl-1-propanol, 2-Methyl-1-butanol, 2-Ethyl-1-hexanol, Benzyl alcohol, Phenethyl alcohol, Terpinen-4-ol, Alpha,2,6,6-tetramethylcyclohexene-1-propan-1-ol	(4) Ethyl acetate, Isoamyl acetate, Linalyl acetate, Phenethyl acetate	(9) Ethyl 2-methylbutyrate, Ethyl isovalerate, Ethyl hexanoate, Ethyl 3-hexenoate, Ethyl caprylate, Ethyl benzoate, Ethyl phenylacetate, Ethyl caprate, Ethyl laurate,	(3) Octanoicacid, 3-Methylbutylester, Isobutyl decanoate, Decanoicacid,3-Methylbutylester
Peach (*Prunus persica* L. Batsch)	(7) 3-Methyl-1-butanol, 2-Methyl-1-propanol, Phenylethyl alcohol, 1-Nonanol, 1-Butanol, 1-Hexanol, 2-Ethyl-1-hexanol	(5) Ethyl acetate, Isoamyl acetate, Isobutyl acetate, Hexyl acetate, Phenethyl acetate	(9) Ethyl lactate, Ethyl butyrate, Ethyl palmitate, Ethyl isovalerate, Ethyl hexanoate, Ethyl nonanoate, Ethyl 2-furoate, Ethyl caprate, Ethyl laurate	Lactones: (2) gamma-Decalactone, delta-Decalatone

Persimmon (*Diospyros kaki* L.)	(4) Propanol, Isobutyl alcohol, Isoamyl alcohol, Phenylethyl alcohol	(2) Ethyl acetate, Isoamyl acetate	(10) Ethyl butyrate, Ethyl valerate, Ethyl caproate, Ethyl caprylate, Ethyl caprate, Ethyl 9-tetradecenoate, Ethyl laurate, Ethyl myristate, Ethyl palmitate, Ethyl palmitoleate	(4) Methyl octanoate, Methyl caprate, Methyl benzoate, Octaethylene glycol monododecyl ether
Pineapple (*Ananas comosus* L. Merr.)	(10) 2-Methyl-1-propanol, 3-Methyl-1-butanol, 2-Methylbutan-1-ol, 2,3-Butanediol, Trans-3-hexen-1-ol, Heptan-1-ol, 2-Ethylhexan-1-ol, 1-Octanol, 2-Phenylethanol, 3,7-Dimethyl-6-octen-1-ol	(5) Ethyl acetate, Isobutyl acetate, Isoamyl acetate, 2-Methylbutyl acetate, 2-Phenethyl acetate	(11) Ethyl acetate, Ethyl propionate, Ethyl isobutyrate, Ethyl butyrate, Ethyl 3-hydroxybutyrate, Ethyl hexanoate, Ethyl benzoate, Ethyl octanoate, Ethyl decanoate, Ethyl laurate, Ethyl myristate	(2) Methyl butyrate, Diethyl succinate
Plum (*Prunus salicina* L.)	(12) Isoamyl alcohol, 2,3-Butanediol, Gentanol, Benzyl alcohol, 1-Octanol, 2-Phenylethanol, 1-Nonanol, Decyl alcohol, 1-Pentadecanol, 5-Methylhexanol, (*E*)-3-Hexen-1-ol, (*Z*)-Hex-4-en-1-ol	(4) Ethyl acetate, Hexyl acetate, Isoamyl acetate, Phenethyl acetate	(11) Ethyl butyrate, Ethyl caproate, Ethyl 2-furoate, Ethyl benzoate, Ethyl octanoate, Ethyl nonanoate, Ethyl caprate, Ethyl laurate, Ethyl palmitate, Ethyl pentadecanoate, Diethyl succinate	(6) Isoamyl decanoate, Methyl myristate, Methyl palmitoleate, Methyl hexadecanoate, Methyl salicylate, Diisobutyl phthalate,
Pomegranate (*Punica granatum* L.)	(8) Methyl-1-propanol, 3-Methyl-1-butanol, 2-Methyl-1-butanol, 2,3-Butanediol, (*Z*)-3-Hexen-1-ol, 1-Hexanol, 2-Ethyl-1-hexanol, 2-Phenylethanol	(5) Ethyl acetate, 3-Methylbutyl acetate, 2-Methylbutyl acetate, Hexyl acetate, 2-Phenylethyl acetate	(10) Ethyl propanoate, ethyl butanoate, Ethyl hexanoate, Ethyl octanoate, Ethyl phenylacetate, Ethyl 9-decanoate, Ethyl decanoate, Ethyl dodecanoate, Ethyl tetradecanoat, Ethyl hexadecanoate	
Ponkan (*Citrus reticulata* Blanco *cv. Ponkan*)	(12) Isobutyl alcohol, Butyl alcohol, 2-Methyl-3-buten-2-ol, 3-Penten-2-ol, Isoamyl alcohol, Amyl alcohol, 2,3-Butanediol, 3-Methyl-1-pentanol, Hexanol, Methionol, Phenethyl alcohol, 4-Hydroxyphenethyl alcohol	(3) Isobutyl acetate, Isoamyl acetate, Phenethyl acetate	(13) Ethyl isobutyrate, Ethyl lactate, Ethyl 3-hydroxybutyrate, Ethyl caproate, Ethyl 4-hydroxybutanoate, Ethyl 2-hydroxycaproate, Ethyl caprylate, Ethyl caprate, Ethyl 3-methylbutyl butanedioate, Ethyl 2-hydroxy-3-phenylpropanoate, Ethyl 2-hydroxypentanedioate, Ethyl linolenate, Ethyl oleate	(10) Methyl tiglate, Methyl lactate, Methyl 3-methoxypropionate, Trans-2-hexenyl butyrate, Diethyl succinate, Methyl linoleate, Methyl linolenate, Methyl oleate, Methyl trans-8-octadecenoate, Methyl linolelaidate

Raspberry (*Rubus idaeus* L.)	Ethanol, 2-Propanol, 1-Propanol, 2-Methyl-1-propanol, 1-Butanol-d, 1-Butanol-M, 3-Methyl-3-buten-1-ol, 3-Methyl-1-pentanol, 1-Hexanol-d, 1-Hexanol-m,	Propyl acetate, Isobutyl acetate, Isoamyl acetate-d, Isoamyl acetate-m, Hexyl acetate,	Ethyl formate, Ethyl acetate, Ethyl propanoate, Ethyl isobutyrate, Ethyl butyrate, Ethyl 2-methylbutanoate, Ethyl isovalerate, Ethyl pentanoate, Ethyl hexanoate-d, Ethyl hexanoate-m, Ethyl heptanoate, Ethyl 2-hexenoate, Ethyl octanoate, Ethyl nonanoate	Methyl 2-methylbutanoate-D, Methyl 2-methylbutanoate-M
Strawberry (*Fragaria* × *ananassa*)	(10) 1-Propanol, 2-Methyl-1-propanol, 1-Butanol, 3-Methyl-1-butanol, 4-Methyl-1-pentanol, 1-Hexanol, 1-Heptanol, 1-Octanol, 3,7-Dimethyl-(*R*)-6-octen-1-ol, Phenylethanol	(3) Ethyl acetate, Hexyl acetate, Phenylethyl acetate	(3) Ethyl hexanoate, Ethyl octanoate, Ethyl decanoate	(3) 3-Methyl-ethyl butyrate, 2-Hydroxy-(*S*)-Ethyl propionate, Diethyl succinate
Wax apple (*Syzygium samarangense*)	(20) Propanol, Isobutanol, 1-Butanol, 3-Methyl-1-butanol, 2-Methylbutan-1-ol, (3S)-3-methylpentan-1-ol, 2,3-Butanediol, 1-Hexanol, Trans-3-hexen-1-ol, cis-4-Hexen-1-ol, 2-Ethylhexan-1-ol, 3,7-Dimethyloct-1,6-dien-3-ol, 1-Octanol, cis-3-Nonen-1-ol, 2-Phenylethanol, L-Menthol, a-Cadinol, 4-Terpineol, a-Terpineol	(8) Ethyl acetate, Isobutyl acetate, Butyl acetate, Isoamyl acetate, cis-3-Hexenyl acetate, Hexyl acetate, 2-Phenethyl acetate, 2-Ethylhexyl acetate	(18) Ethyl propionate, Ethyl isobutyrate, Ethyl butyrate, Ethyl lactate, Ethyl 2-methylbutyrate, Ethyl isovalerate, Ethyl hexanoate, Ethyl trans-4-decenoate, Ethyl octanoate, Ethyl 3-hydroxybutyrate, Ethyl nonanoate, Ethyl decanoate, Ethyl benzoate, Ethyl phenylacetate, Ethyl stearate, Ethyl laurate, Ethyl myristate, Ethyl hexadecanoate	(3) Methyl octanoate, 3-Methylbutyl octanoate, Diethyl succinate

Note: The numbers in parentheses refer to the number of volatile compounds of the same group.

Continued supplementary table 1 Fruits and their volatile compounds in fruit wines

Fruits	Aldehydes, ketones and minor volatiles	Terpenes and other volatiles	Acids	Yeasts and lactic acid bacteria	Ref.

Apple (*Malus domestica* Borkh.)	(4) Benzaldehyde, 3-Hydroxy-β-damascone, 3-Oxo-α-Ionolo, β-Damascenone	(3) Linalool, Geraniol, 4-Vinyl guaiacol	L-malic acid, Acetic acid, Isovaleric acid, Hexanoic acid, Octanoic acid	*Saccharomyces cerevisiae* EC1118, *S. uvarum*, *Torulaspora delbrueckii* TD291, *Hanseniaspora osmophila*, *H. uvarum*, *Starmerella bacillaris* and *Zygosaccharomyces bailii*,	Lorenzini et al. 2019
Apple (*Malus domestica* Borkh.)	(3) Benzaldehyde, Nonanal, 2-Butanone	Terpenoids (3) Linalool oxide (furanoid), Linalool, α-Farnesene	(3) 3-Methyl butanoic acid, 2-Methyl butanoic acid, Hexanoic acid, Octanoic acid	*Saccharomyces cerevisiae* var. *Bayanus* EC-1118	Ruppert et al. 2021
Apricot (*Prunus armeniaca* L.)		Linalool, *α*-Terpineol Lilac, Citronellol, Geraniol Citric	Citric acid, Tartaric acid, Malic acid, Quinic acid, Succinic acid, Acetic acid	*S. cerevisiae* var. *Bayanus* EC-1118	Choi et al. 2020
Bilberry (*Vaccinium myrtillus* L.)	Aldehydes: (6) Acetaldehyde, 3-Methylbutanal, Hexanal, (E)-2-Hexenal, Nonanal, Benzaldehyde<br>Ketones: (6) 2-Pentanone, 4-Methyl-2-pentanone, 4,6-Dimethyl-2-heptanone, Acetoin, 6-Methyl-5-hepten-2-one, 2,6,8-Trimethyl-4-nonanone	Monoterpenes: (3) Linalool, α-Terpineol, β-Citronellol<br>Acetals: (5) 1-Ethoxy-1-methoxyethane, 1,1-Diethoxyethane, 2,4,5-Trimethyl-1,3-dioxolane, 2,4-Dimethyl-1,3-dioxane, 1-(1-Ethoxyethoxy)-pentane<br>Benzenes: (2) 1,3,5-Trimethylbenzene, 1,3-Di-tert-butylbenzene		*Saccharomyces cerevisiae* 1116, *Schizosaccharomyces pombe* 3796, *Schizosaccharomyces pombe* 70572, *Torulaspora delbrueckii* 291, and *T. brueckii* 70526	Liu et al. 2019
Black raspberry (*Rubus occidentalis* L.)	Aldehydes: (5) Acetaldehyde, Propionaldehyde, butanal, Nonanal, Methional<br>Ketones: (5) Acetone, 2-Pentanone, 2,3-Butanedione, 2-Octanone, 3-Hydroxy-2-butanone	α-Terpinene, Terpinolen		*S. cerevisiae* Lalvin RHST, T. delbrueckii Viniflora Prelude™, *O. oeni* Viniflora Oenos	Liu et al. 2020

Blueberry (*Ericaeae Vaccinium*)		(17) 1,8-Cineoleommon, (6*E*) -Nerolidol, β-Ocimene, Trans- / *β*-ocimene, *o*-Cymene, *α*-Campholenal, Terpinolene, Limonene, *α*-Terpinene, Myrcenol, Nerol oxide, Linalool, Geraniol, *β*-Terpineol, Cis-linalool oxide, Linalool oxide, Norisoprenoids (*E*)-Geranylacetone	(6) Acetic acid, Decanoic acid, Nonanoic acid, Dodecanoic acid, Butanoic acid, Isovaleric acid	*Tourlaspora delbrueckii* Zymaflore ® Alpha and *Saccharomyces cerevisiae* Actiflore F33	Wang et al. 2023
Blueberry (*Vaccinium corymbosum* L.)	(7) Acetaldehyde, Syringaldehyde, Acetoin, 3-Hydroxy-7,8-dihydro-*β*-ionone, 3-oxo-α-ionol, 3-Hydroxy-7,8-dihydro-*β*-ionol, Methionol	(12) 8-Hydroxy-6,7-dihydrolinalool, *E*-8-Hydroxylinalool, *Z*-8-Hydroxylinalool, Linalool, α-Terpineol, 4-Vinylguaiacol, Eugenol, Linalool hydrate, cis-Furan linalool oxide, *β*-Citronellol, trans-Carveol, *p*-1-Menthen-9-ol	(10) Acetic acid, Citric acid, Tartaric acid, Malic acid, Succinic acid, Lactic acid, Hexanoic acid, Octanoic acid, 2-Methylpropanoic acid, 3-Methylbutyric + 2-Methylbutyric acids	*Saccharomyces cerevisiae* Lalvin QA23	Mendes-Ferreira et al. 2019
Kiwi fruit (*Actinidia chinensis*)	Aldehydes: (7) Hexanal, trans-2-Hexenal, Heptanal, Benzaldehyde, 3,5-Dimethylbenzaldehyde, Nonanal, Decanal Ketones: (7) 2-Octanone, 3-Pentanone, 2-Heptanone, 6-Methyl-5-hepten-2-one, 3-Octanone, 2-Nonanone, 2-Undecanone	Terpenes (6) Terpinen-4-ol, Camphene, *γ*-Terpinene, Bornylene, Cineole, α-Cubebene	(16) Oxalic acid, Tartaric acid, Pyruvic acid, Malic acid, Shikimic acid, Lactic acid, Acetic acid, Citric acid, Succinic acid, Isobutyric acid, Butanoic acid, Isovaleric acid, 2-Methylbutyric acid, Hexanoic acid, Octanoic acid	*S. cerevisiae* WLS21, *Wickeramomyces anomala* BLCC12, *Zygosaccharomyces rouxii* IFO30, *Z. bailii* IFO37, and *Schizosaccharomyces pombe* 1757	Li et al. 2022b
Longan (*Dimocarpus longan* Lour.)	(8) 1-Nonanal, Decanal, Benzaldehyde, 2-Ethylidene-6-methyl-3,5-heptadienal, (*E*)-2-Decenal, trans-2-Undecenal, 3-Hydroxy-2-butanone, 2-Undecanone, 2-	(8) Linalool oxide, *β*-Citronellol, Linalool, l-Limonene, (*E*)-3,7-Dimethyl-1,3,6-octatriene, 2,6-Dimethyl-2,4,6-octatriene, trans-1,1,3,5-	Acetic acid	*Saccharomyces cerevisiae* F33, Lalvin 71B, Lalvin EC1118, Lalvin D254, Lalvin RC212, Lalvin RC2323, Lalvin K1, Lalvin U43,	Liu et al. 2018

	Bromo-4-chloroanisole, 2-Methyl-1-nonene-3-yne,	Tetramethyl-cyclohexane, Decamethylcyclopentasiloxane		Lalvin KD and Lalvin R-HST, *S. cerevisiae* Angel BV818, Angel SY and Angel RW	
Mango (*Mangifera indica* L.)	Aldehydes: (2) *β*-Cyclocitraln, sophthalaldehyde Ketones: (3) Isophorone, (*E*)-Geranylacetone, β-Ionone	Alkenes: (9) Cyclofeuchene, Myrcene, *α*-Phellandrene, Delta.3-Carene, *α*-Terpipene, DL-Limonene, *α*-Terpinolene, trans-Caryophyllene, α-Humulene Alkanes: (2) Dodecane, Tetradecane others: (4) Hexeadecane, 2-Methylbutanoic acid, *p*-Cymene, Naphthalene, Linalool	Citric Acid, Oxalic acid, Malic acid, Succinic acid, *α*-Ketoglutaric acid, Tartaric acid	*Pichia kudriavzevii* HDX1, *P. kudriavzevii* HDY1, and *P. kudriavzevii* HDA2	Bao et al. 2021
Mulberry (*Morus* spp.)	Aldehydes: (8) Acetaldehyde, Propanal, Pentanal, Hexanal, 2-Hexenal, Benzaldehyde, Methional, (*E*, *E*)-2,4-Hexadienal; Ketones: (3) Acetone, Cyclopentanone, Acetoin	(7) Linalool, *α*-Pinene, *α*-Terpinolene, *γ*-Terpinene, *p*-Cymene, *p*-Xylene, Dimethyl sulfide	(3) Acetic acid, Propanoic acid, Butanoic acid	*S. cerevisiae* GRE, *O. oeni* of Viniflora ® CH11, and *Lb. plantarum* of Bactoferm ® vege-start 2.0 CN	Zhang et al. 2022b
Orange (*Citrus sinensis* Osb. var. *brasiliensis* Tanaka)	(2) 4-Hydroxy-5-methyl-3(2*H*)-furanone, 2-Methoxy-4-vinylphenoll	(4) Linalool, Carveol, Citronellol, cis-Carveol	(9) Tartaric acid, Malic acid, Ascorbic acid, Lactic acid, Acetic acid, Citric acid, Succinic acid, Isovaleric acid, Decanoic acid	*S. cerevisiae* Sc NCUF309.2, *T. delbrueckii* Td NCUF305.2	Liu et al. 2023
Passion fruit (*Passiflora edulis*)	Ketones: (2) Sulcatone, Nonanal Benzenes: (2) Styrene, Benzaldehyde Phenols: (2) 4-Vinylguaiacol, 2,4-Di-tert-butylphenol	Terpenes: (24) *β*-Myrcene, *D*-Limonene, *β*-Ocimene, Terpinolen, Neo-allo, 4-Terpineol, *α*-Terpineol, Linalool, Citronellol, Nerol, Nerolidol, Geranyl acetone, cis-Rose oxide, trans-Rose oxide, Geraniol, cis-Furan linalool	(7) Isobutyric acid, Isovaleric acid, Hexanoic acid, Octanoic acid, Decanoic acid, 9-Decenoic acid, Lauric acid	*Saccharomyces cerevisiae* ES488 and CY3079, *Saccharomyces bayanus* BV818 and VIC	Liu et al. 2022a

		oxide, trans-Furan linalool oxide, Norisoprenoids, *β*-Damascone, *α*-Ionone, *β*-Ionone, Edulan I, Edulan II, Furan, 2,3-Dihydro-benzofuran<br>Sulfur Volatiles: (15) Benzothiazole, 4-Methyl-5-vinylthiazole, Thiazole, 2-Methyl-4-propyl-1,3-oxathiane, 2-Methyl-1,3-oxathiane, Diethyl disulfide, Ethyl 3-(methylthio)-(*E*)-2-propenoate, 3-(Methylthio)propyl acetate, Methionol, 3-Mercaptohexanol, 3-Mercaptohexyl hexanoate, 3-Mercaptohexyl acetate, Diisopropyl disulfide, Ethanethiolic acid, [2-(Ethylsulfanyl) ethyl]benzene, 3-(Methylthio)-2-butanone			
Peach (*Prunus persica* L.)	(9) Benzaldehyde, 1-Nonanal, *β*-Damascone, *γ*-unsecalactone, Hexamethylcyclotrisiloxane, Octamethyl cyclotetrasiloxane, Theaspirane, 2-Methoxy-6-[(*E*)-prop-1-enyl]phenol, Hexadecamethylheptasiloxane		Acetic acid, Hexanoic acid, Octanoic acid, Decanoic acid	*Saccharomyces cerevisiae* PY01	Liu er al. 2020a
Peach (*Prunus persica* L. Batsch)		(2) Linalool, (*R*)-(+)-beta-citronellol	Organic acids (g/L): (8) Oxalic acid, Tartaric acid, Quinic acid, Pyruvic Acid, Malic acid, Lactic acid, Citric acid, Succinic acid<br>Fatty acids: (3) Hexanoic acid,	*Saccharomyces cerevisiae* WT-21, *Hanseniaspora uvarum* #15 and *Metschnikowia pulcherrima* #30, and *Lachancea thermotolerans* Concerto, *Torulaspora delbrueckii* Zymaflore® AlphaTD N. SACCH	Liu et al. 2022b

			Octanoic acid, Decanoic acid, Dodecanoic acid		
Persimmon (*Diospyros kaki* L.)	(5) Acetaldehyde, Nonanal, 2-Furaldehyde, Naphthalene, 2,4-Ditertiary butyl phenol		(4) Acetic acid, Octanoic acid, Decanoic acid, Lauric acid		Nie et al. 2023
Pineapple (*Ananas comosus* L. Merr.)	(8) Acetaldehyde, Acetal, Benzaldehyde, Octanal, Phenylacetaldehyde, Acetophenone, Nonanal, Decanal	Sulphur compounds: (4) 3-Methylthiopropanol, Methyl 3-methylthiopropionate, Ethyl 3-methylthiopropionate, Dihydro-2-methyl-3(2*H*)-thiophenone<br>Styryl derivatives: (2) Styrene, 2,4-Dimethylstyrene; Terpenes: Limonene<br>Furans: (3) 2-Methylfuran, 2,4-Dimethylfuran, 4-Hydroxy-2,5-dimethylfuran-3-one	(7) Acetic acid, Isobutyric acid, Isovaleric acid, 2-Methylbutyric acid, Hexanoic acid, Octanoic acid, Decanoic acid	*S. cerevisiae* D254, VIC, BV818 (var. *bayanus*), and CECA	Lin et al. 2018
Plum (*Prunus salicina* L.)	(3) Phenylacetaldehyde, Phenyl *p*-tolyl ketone, 3-Methylthiopropanol	(5) (+)-Limonene, Linalool, Citronellol, l-Caryophyllene, Nerolidol	(11) Acetic acid, Hexanoic acid, Octanoic acid, Decanoic acid, Oxalic acid, Tartaric acid, Pyruvic acid, Malic acid, Lactic acid, Citric acid, Succinic acid	*S. cerevisiae* (D254), *Metschnikowia pulcherrima*,	Zhang et al. 2022a
Pomegranate (*Punica granatum* L.)		(3) Eucalyptol, *α*-Terpineol, Trans-nerolidol	(4) Octanoic acid, Decanoic acid, Dodecanoic acid, Hexadecanoic acid	*Saccharomyces bayanus* SN9, *Saccharomyces cerevisiae* M02-Cider, and *Saccharomyces cerevisiae* var. *diastaticus* SAFALE™ WB-06	Kokkinomagoulos et al. 2020

Ponkan *Citrus reticulata* Blanco *cv. Ponkan*	(5) 3-Hydroxy-2-butanone, Butyrolactone, Geranyl acetone, a-Ionone, Farnesyl acetone	(18) Elemol, Farnesol, *t*-Muurolol, *b*-Eudesmol, *a*-Cadinol, *a*-Pipene, *g*-Terpinene, Limonene, *a*-Terpineol, *d*-Cadinene, *a*-Calacorene, Cadina-1,4-diene, *a*-Cubebene, *g*-Elemene, *a*-Cedrene, *a*-Copaene, 2,6-Dimethoxy-4-allylphenol, 4-Methyl-2,6-di-tert-butylphenol, 2,4-Di-tert-butylphenol	(12) Acetic acid, Hexanoic aci, Benzoic acid, Caprylic acid, Nonoic acid, Capric acid, Lauric acid, Myristic acid, 9-Hexadecenoic acid, Palmitic acid, Octadecanoic acid, Linoleic acid	HF-8, BCRC 21761, BCRC 21805, BCRC 21823, BCRC 22293, and BCRC 22332,	Lee et al. 2013
Raspberry (*Rubus idaeus* L.)	(7) Acetaldehyde, Propionaldehyde, Nonanal, Methional, Acetone, 2-Pentanone, 2-Octanone	*α*-Terpinene	Acetic acid	*S. cerevisiae* L' Authentiqu, *Torulaspora delbrueckii* Biodiva	Li et al. 2020
Strawberry (*Fragaria* × *ananassa*)	(1) Benzaldehyde		(3) Acetic acid, 2-Methyl-propanoic acid, Octanoic acid	*Saccharomyces cerevisiae* strain LALVIN CY3079™	Feng et al. 2015
Wax apple (*Syzygium samarangense*)	(7) Acetaldehyde, Acetal, Furfural, Decanal, Phenylacetaldehyde, Nonanal, 2-Nonanone	Sulphur compounds: (3) Dimethyl sulfide, 3-Methylthiopropanol, Dihydro-2-methyl-3(2*H*)-thiophenone; Styryl derivatives: (1) Styrene; Lactones: (1) *c*-Butyrolactone; Terpenes: (2) Limonene, *p*-Cymene; Furan: (2) 2-Ethylfuran, 2,3-Dihydrobenzo	(8) Acetic acid, Isobutyric acid, Butyric acid, Isovaleric acid, 2-Methylbutyric acid, Hexanoic acid, Octanoic acid, Decanoic acid	*Saccharomyces cerevisiae* D254, VIC, BV818, and RV100	Lin et al. 2019

Note: The numbers in parentheses refer to the number of volatile compounds of the same group.
